# Prognostic factors in the treatment of squamous cell carcinoma of the larynx: partial surgery x radical surgery

**DOI:** 10.1016/S1808-8694(15)31178-2

**Published:** 2015-10-19

**Authors:** Maria da Graça Caminha Vidal, Onivaldo Cervantes, Marcio Abrah ão, Flávio Carneiro Hojaij, Ali Amar

**Affiliations:** 1 Graduate student from the Escola Paulista de Medicina SP, Head of the SCCP - Hospital da Universidade Federal de Santa Maria - RS; 2 Professor of the Head and Neck Surgery Department /Otorhinolaryngology of the Escola Paulista de Medicina de São Paulo, Head of the SCCP of the Escola Paulista de Medicina - SP; 3 Professor of the Head and Neck Surgery Department /Otorhinolaryngology of the Escola Paulista de Medicina de São Paulo, Head physician of the SCCP of the Escola Paulista de Medicina - SP; 4 PhD in Head and Neck Surgery from the Hospital das Clínicas - Faculdade de Medicina da Universidade de São Paulo, Assistant Physician of the SCCP of the Escola Paulista de Medicina; 5 PhD in Head and Neck Surgery from the Escola Paulista de Medicina de São Paulo, Assistant Physician at the Hospital Heliópolis São Paulo

**Keywords:** larynx, surgery, cancer, prognosis, survival, treatment

## Abstract

The present study aimed at assessing the other sites as the carcinoma spreads, as well as therapeutic results, in larynx preservation and patient survival. **Study type**: It is a Longitudinal Historical Cohort Study, a retrospective clinical study.

**Materials and Methods:**

A hundred and sixty (160) patients treated at 'Escola Paulista de Medicina' ('Paulista' Medical School) - São Paulo Hospital, from January 1988 to December 2004 were examined as for the spreading of their larynx carcinoma. Those patients whose evaluations were at least two years old after treatment were the only ones accepted. The parametric tests used were: Test X2, Fisher's test, and Kaplan-Meier's curve.

**Results:**

The posterior commissure and the infraglottis were significant in terms of the laryngectomy: glottal tumors AC: (p=0.03) AP: (p=0.0001); AC: (p=0.0007) AP: (p<0.0001), respectively. The infraglottis was significant in G+SG tumors in AP: (p=0.04) and in death rate AP: (p=0.03).

**Conclusion:**

total laryngectomy is the treatment of choice in the presence of total involvement of the posterior commissure and the infraglottis. The latter may compromise survival, according to local invasion, even in the presence of free surgical margins.

## INTRODUCTION

It is important to understand the dissemination of endolaryngeal carcinoma for staging and treatment aiming at locoregional control and survival.^1^,^2^

The need to study the internal structures or subsites of the larynx (anterior commissure, posterior commissure, ventricle and infraglottis) since their origin, evolution, and their relation with the invasion and barrier paths to the tumor have been well documented in the literature.^1^, ^2^, ^3^, ^4^, ^5^, ^6^, ^7^, ^8^, ^9^, ^10^, ^11^, ^12^, ^13^, ^14^, ^15^, ^16^ The objective of this study was to assess the laryngeal regions and subsites of 160 patients with laryngeal carcinoma, in the period between January 1998 and December 2004. Moreover, it aimed to check the proportion of affected subsites and their influence on determining conservative or radical surgery (total laryngectomy), as well as the repercussion on local control, organ preservation and survival.

This research was analyzed and approved under No. CEP 1610/04 by the Research Ethics Committee of the Universidade Federal de São Paulo.

## MATERIALS AND METHODS

First, a total of 200 medical charts of patients with laryngeal carcinoma were reviewed. Patients were seen at the Escola Paulista de Medicina - Hospital São Paulo, in the period from January 1998 to December 2004. The tumors were classified according to the TNM and AJCC/UICC systems (2002).

Next, a database was created for patients with initial indication for surgical treatment.

The main objective was to assess the involvement level of the internal structures or subsites (anterior commissure, posterior commissure, ventricle and infraglottis) by the neoplasm. Furthermore, to evaluate the repercussion of these structures affected by the tumor have in treatment, local control, organ preservation, and survival.

These structures were studied through specific clinical examinations, including videotelelaryngoscopy, image exams such as computerized tomography of the larynx, and endoscopic examination (nasal fibrobronchoscopy). All exams required for diagnosis, staging and preoperative evaluation were also performed. Biopsy was performed through microsurgery of the larynx.

Inclusion Criteria for the Database: Patients with full description of structure involvement, through clinical assessment, pathologic evaluation, diagnosis, staging, treatment, condition of surgical margins, and progression in the referenced time, patient's condition with information updated and recorded until December 2004.

Exclusion Criteria for the Database: Patients with incomplete data, no updated record, follow-up for less than two years after diagnosis and treatment, or loss of control. Patients with inaccurate description of internal structure involvement, or with incomplete evaluations, either by clinical, endoscopy, or image exams. Patients who underwent surgery in 2004 were also excluded since they had not been followed-up for at least two years during the referenced period.

According to these criteria, 160 patients were selected for the database. However, 17 patients from this group, initially with surgical indication, went to radiotherapy and/or chemotherapy due to clinical conditions or refusal to surgery. Only two patients were submitted to microsurgery of the larynx to diagnose and assess the endolarynx, without tracheostomy: one T1 patient and one T2 patient. The other 15 patients were six T4 patients and nine T3 patients, who underwent tracheostomy, in addition to microsurgery of the larynx. They were included in the initial database for having full description of the subsites affected by the tumor, besides other items. In this case, the pathologic evaluation and the clinic evaluation were considered the same, since there was no change in the results.

The remaining 143 patients in the database went to surgical treatment: partial or total laryngectomy according to previous indication, staging, as well as preoperative and intraoperative presentation of the neoplasm.

Patients with the disease after clinical or surgical treatment had the same value as patients who died due to the disease, in the period in which survival was studied.

The mean age was 58.9 years. The predominant sex was male, 87.5%. Dysphonia was the main symptom, with 79.4%, lasting 11.9 months in average. A total of 89.4% of patients were smokers and 50% suffered from alcoholism.

The research was developed by a longitudinal historical cohort study (retrospective clinical study). The nonparametric tests used were X2 test, Fisher test and the Kaplan-Meier curve, and the significance level was 5%.

## RESULTS

Most laryngeal carcinomas presented originated from or were located in the glottis (CA: n=105 and PA: n=100), according to the following results:

List of patients (n) with origin or location of the tumor in the larynx.

**Figures 1 and 2:**
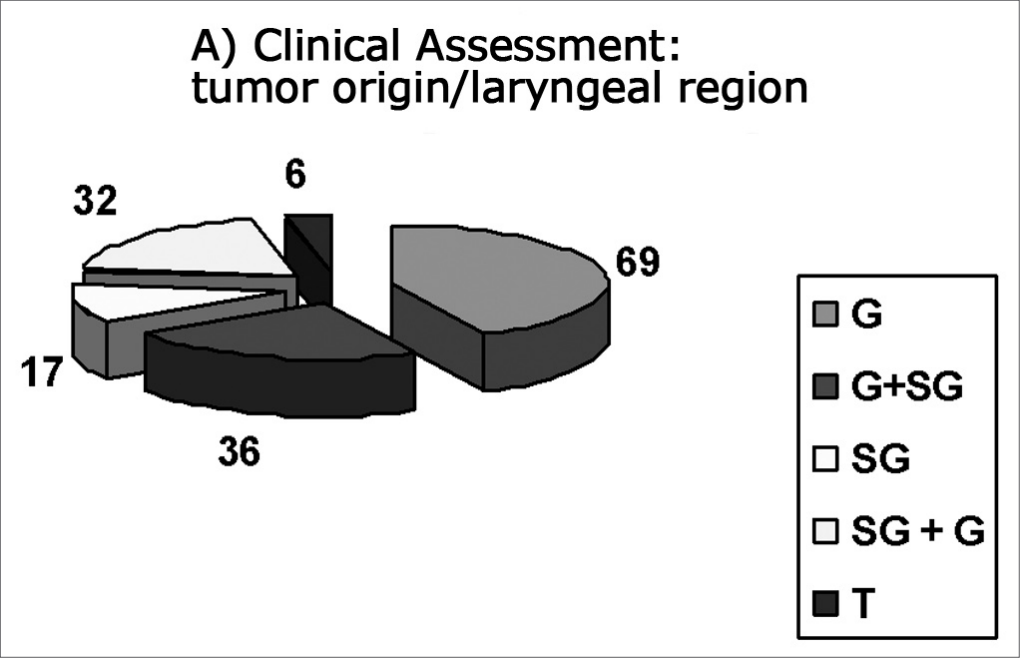
CA: Clinical assessment: number of patients with laryngeal regions involved by the tumor PA: Pathological assessment: number of patients with laryngeal regions involved by the tumor G: Glottic Tumors (CA: 69 patients, PA: 65 patients) G + SG: Glottic tumors extending to the supraglottis (CA: 36 patients, PA: 35 patients) SG: Supraglottic tumors (CA: 17 patients, PA: 15 patients) SG + G: Supraglottic tumors extending to the glottis (CA: 32 patients, PA: 31 patients) T: Transglottic tumors (CA: 6 patients, PA: 14 patients) Note: In tumors (T): in CA: 5 patients with glottic origin and 1 patient with supraglottic origin. In PA: 10 patients with glottic origin and 4 patients with supraglottic origin.

This list is shown in Figures 1 and 2.

Charts 1 and 2 display tumor staging and the relation of origin in the larynx (glottic or supraglottic) in clinical and pathological assessment, respectively.

Proportion of involved subsites in relation to tumor site and type of surgery - conservative vs. total

Tables 1 and 2 show the involvement degree of the anterior commissure due to the neoplasm, by clinical and pathological assessment between conservative laryngectomy and total laryngectomy.

Tables 3 and 4 show the involvement degree of the posterior commissure due to the neoplasm, by clinical and pathological assessment between conservative laryngectomy and total laryngectomy.

Tables 7 and 8 show the involvement degree of the infraglottis due to the neoplasm, by clinical and pathological assessment between conservative laryngectomy and total laryngectomy.

Tables 1 to 8.

G: Glottic tumors G+SG: Glottic tumors extending to the supraglottis

SG: Supraglottic tumors SG+G: Supraglottic tumors extending to the glottis

**Chart 1:** Glottic origin tumor (T) staging in clinical and pathological assessment.

TUMOR (T)	CLINICAL ASSESSMENT			PATHOLOGICAL ASSESSMENT		
	G	G+SG	T	G	G+ SG	T
	n %	n %	n %	n %	n %	n %
T1a	12 17.3	0 0.0	0 0.0	12 18.4	0 0.0	0 0.0
T1b	19 27.5	1 2.7	0 0.0	20 30.7	0 0.0	0 0.0
T2	17 24.6	8 22.2	0 0.0	13 20.0	6 17.1	0 0.0
T3	13 18.8	18 50.0	0 0.0	11 16.9	15 42.8	0 0.0
T4	8 11.5	9 25.0	5 100.0	9 13.8	14 40.0	10 100.0
TOTAL	69 100.0	36 100.0	5 100.0	65 100.0	35 100.0	10 100.0

**Chart 2:** Supraglottic origin tumor (T) staging in clinical and pathological assessment.

TUMOR (T)	CLINICAL ASSESSMENT			PATHOLOGICAL ASSESSMENT		
	SG	SG+G	T	SG	SG+G	T
	n %	n %	n %	n %	n %	n %
T1	3 17.6	0 0.0	0 0.0	2 13.3	1 3.22	0 0.0
T2	7 41.1	3 9.37	0 0.0	6 40.0	5 16.1	0 0.0
T3	4 23.5	18 56.2	0 0.0	3 20.0	16 51.6	0 0.0
T4	3 17.6	11 34.3	1 100.0	4 26.6	9 29.0	4 100.0
TOTAL	17 100.0	32 100.0	1 100.0	15 100.0	31 100.0	4 100.0

AC: Anterior commissure PC: Posterior commissure or interarytenoid space

V: Ventricle IG: Infraglottis

W/O: Absence of subsite involvement

With: Presence of subsite involvement

N: Absolute number of cases %: Percentage corresponding to (N) of cases

C.L.: Conservative laryngectomy

T. L: Total laryngectomy

Chart 4 shows the number of conservative surgeries in relation to tumor (T) extension in the larynx.

**Chart 4:** Frequency distribution of conservative surgery in relation to the tumor (T).

Tipos de Cirurgias Conservadoras								
Tumor (T)	Chordectomy	Frontolateral Laryngectomy	Hemilaryngectomy	PSCL CHEP II	PSCL CHEP I	PSCL CHP	HSGL	TOTAL
T1a	8*	*3	0	1	0	0	2	14
T1b	4*	*12*	2	1	1	0	1	21
T2	1	*9	2	8*	5	2	3*	30
T3	0	0	0	1	2	*3	0	6
Total	13	24	4	11	8	5	6	71

*Four patients with involved margins: 3 patients: submitted to: Frontolateral Laryngectomy: T1a, T1b, T2. 1 patient: submitted to: LHSC-CHP: T3.

All were referred to R T, only one patient died (disease).

*Partial surgery converted to total: 4 patients due to local recurrence and one by chronic aspiration (HSGL).

*Four patients with involved margins: 3 patients: submitted to: Frontolateral Laryngectomy: T1a, T1b, T2. 1 patient: submitted to: LHSC-CHP: T3. All were referred to RT, only one patient died (disease).

*Partial surgery converted to total: 4 patients due to local recurrence and one by chronic aspiration (HSGL).

Tables 9 and 10 show the number of deaths in relation to the subsite that interfered with survival according to its degree of involvement in clinical and pathological assessment.

**Chart 3:** Statistical distribution of the type of treatment in relation to the tumor (T).

Type of Surgery										
Tumor (T)	No surgery n %	C n %	PFLL n %	HL n %	HSGL n %	PSCL CHEP II n %	PSCL CHEP I n %	PSCL CHP n %	TL n %	TOTAL n %
T1a	1 0,62	8 4,96	3 1,86	0 0,0	2 1,24	1 0,62	0 0,0	0 0,0	0 0,0	15 9,3
T1b	0 0,0	4 2,51	12 7,54	2 1,25	1 0,62	1 0,62	1 0,62	0 0,0	0 0,0	21 13,2
T2	1 0,62	1 0,62	9 5,60	2 1,24	3 1,86	8 4,98	5 3,11	2 1,24	4 2,49	35 21,8
T3	9 5,62	0 0,0	0 0,0	0 0,0	0 0,0	1 0,62	2 1,25	3 1,87	37 23,1	52 32,5
T4	6 3,76	0 0,0	0 0,0	0 0,0	0 0,0	0 0,0	0 0,0	0 0,0	31 19,43	37 23,2
Total	17 10,6	13 8,12	24 15	4 2,5	6 3,75	11 6,87	8 5	5 3,12	72 45	160 100

**Key**:

C: Chordectomy

PFLL: Partial frontolateral laryngectomy

HL: Hemilaryngectomy

HSGL: Horizontal supraglottic laryngectomy

PSCL: Partial supracricoid laryngectomy

CHEP II: Reconstruction after type II cricohyoidoepiglottopexy

CHEP I: Reconstruction after type I cricohyoidoepiglottopexy

CHP: Reconstruction after type II cricohyoidopexy

TL: Total laryngectomy

No surgery: 17 patients only underwent larynx microsurgery and tracheostomy, except two patients (T1 and T2, respectively) who had only larynx microsurgery. All patients were referred to radiation therapy and/or chemotherapy. One patient (T1) and five patients (T3) survived with no disease, the remaining (n=11) had a negative course of the disease.

**Table 1:** Statistical distribution of the anterior commissure in the site of neoplasm vs. type of laryngectomy - clinical assessment.

ANTERIOR COMMISSURE (AC)																
	G				G+SG				SG				SG+G			
	No		Involvement		No		Involvement		No		Involvement		No		Involvement	
Type	N %		N %		N %		N %		N %		N %		N %		N %	
C.L.	19	70,4	33	75,0	3	23,1	9	40,9	12	80,0	0	0,0	5	31,3	10	33,3
T.L.	8	29,6	11	25,0	10	76,9	13	59,1	3	20,0	0	0,0	11	68,8	20	66,7
TOTAL	27 100,0 44 100,0				13 100,0 22 100,0				15 100,0		0	0,0	16	100,0	30 100,0

**Table 2:** Statistical distribution of the anterior commissure in the site of neoplasm neoplasm vs. type of laryngectomy - clinical assessment.

ANTERIOR COMMISSURE (AC)																
	G				G+SG				SG				SG+G			
	No		Involvement		No		Involvement		No		Involvement		No		Involvement	
Type	N %		N %		N %		N %		N %		N %		N %		N %	
C.L.	25	86,2	24	64,9	5	38,5	11	47,8	5	71,4	6	100,0	18	94,7	13 100,0	
T.L.	4	13,8	13	35,1	8	61,5	12	52,2	2	28,6	0	0,0	1	5,3	0	0,0
TOTAL	27 100,0 44 100,0				13 100,0 22 100,0				15 100,0		0	0,0	16	100,0	30 100,0

**Table 3:** Statistical distribution of the posterior commissure in the site of neoplasm vs. type of laryngectomy - clinical assessment.

POSTERIOR COMMISSURE (PC)																
	G				G+SG				SG				SG+G			
	W/O		With		W/O		With		W/O		With		W/O		With	
Type	N %		N %		N %		N %		N %		N %		N %		N %	
C.L.	46	79,3	6	46,2	10	43,5	2	16,7	11	84,6	1	50,0	5	23,8	4	36,4
T.L.	12	20,7	7	53,8	13	56,5	10	83,3	2	15,4	1	50,0	16	76,2	7	63,6
TOTAL	58	100,0	13	100,0	23	100,0	12	100,0	13	100,0	2	100,0	21	100,0	11	100,0

**Table 4:** Statistical distribution of the posterior commissure in the site of neoplasm vs. type of laryngectomy - pathological assessment.

POSTERIOR COMMISSURE (PC)																
	G				G+SG				SG				SG+G			
	W/O		With		W/O		With		W/O		With		W/O		With	
Type	N %		N %		N %		N %		N %		N %		N %		N %	
C.L.	48	82,8	1	12,5	13	54,2	3	25,0	9	81,8	2	100,0	9	36,0	1	16,7
T.L.	10	17,2	7	87,5	11	45,8	9	75,0	2	18,2	0	0	16	64,0	5	83,3
TOTAL	58 100,0 8 100,0				24 100,0 12 100,0				25 100,0		6 100,0		25 100,0		6 100,0

**Table 5:** Statistical distribution of the ventricle in the site of neoplasm vs. type of laryngectomy - clinical assessment.

VENTRICLE (V)																
	G				G+SG				SG				SG+G			
	W/O		With		W/O		With		W/O		With		W/O		With	
Type	N %		N %		N %		N %		N %		N %		N %		N %	
C.L.	48	76,2	4	50,0	9	34,6	3	33,3	12	80,0	0	0,0	8	34,8	1	11,1
T.L.	15	23,8	4	50,0	17	65,4	6	66,7	3	20,0	0	0,0	15	65,2	8	88,9
TOTAL	63	100,0	8	100,0	26	100,0	9	100,0	15	100,0	0	0,0	23	100,0	9	100,0

**Table 6:** Statistical distribution of the ventricle in the site of neoplasm vs. type of laryngectomy

VENTRICLE (V)																
	G				G+SG				SG				SG+G			
	W/O		With		W/O		With		W/O		With		W/O		With	
Type	N %		N %		N %		N %		N %		N %		N %		N %	
C.L.	43	79,6	6	50,0	13	46,4	3	37,5	11	84,6	0	0,0	9	39,1	1	12,5
T.L.	11	20,4	6	50,0	15	53,6	5	62,5	2	15,4	0	0,0	14	60,9	7	87,5
TOTAL	54 100,0 12 100,0				28 100,0 8 100,0				13 100,0		0 0,0		23 100,0		8 100,0

**Table 7:** Statistical distribution of the infraglottis in the site of neoplasm vs. type of laryngectomy - clinical assessment.

INFRAGLOTTIS (IG)																
	G				G+SG				SG				SG+G			
	W/O		With		W/O		With		W/O		With		W/O		With	
Type	N %		N %		N %		N %		N %		N %		N %		N %	
C.L.	49	81,7	3	27,3	11	40,7	1	12,5	12	80,0	0	0,0	9	28,1	0	0,0
T.L.	11	18,3	8	72,7	16	59,3	7	87,5	3	20,0	0	0,0	23	71,9	0	0,0
TOTAL	60 100,0 11 100,0				27 100,0 8 100,0				15 100,0		0 0,0		32 100,0		0 0,0

**Tabela 8:** Distribuição estatística da infraglote na localização neoplásica x tipo de laringectomia - avaliação patológica.

INFRAGLOTTIS (IG)																
	G				G+SG				SG				SG+G			
	W/O		With		W/O		With		W/O		With		W/O		With	
Type	N %		N %		N %		N %		N %		N %		N %		N %	
C.L.	47	87,0	2	16,7	11	52,4	2	13,3	11	84,6	0	0,0	10	32,3	0	0,0
T.L.	7	13,0	10	83,3	10	47,6	13	86,7	2	15,4	0	0,0	21	67,7	0	0,0
TOTAL	54 100,0	12	100,0	21	100,0	15	10,0	13	100,0	0	0,0	31	100,0	0	0,0

**Table 9:** Statistical distribution of cases of death in the presence or absence of infraglottis involvement - clinical assessment.

Survival							
		Alive		Dead		Total	
		N	%	N	%	N	%
Infraglottis	0	125	85,6%	11	78,6%	136	85,0%
Clinical aspect	1	21	14,4%	3	21,4%	24	15,0%
Total		146	100,0%	14	100,0%	160	100,0%

Chi-square test: (p) = 0.754

**Table 10:** Statistical distribution of cases of death in the presence or absence of infraglottic involvement - pathological assessment.

Survival							
		Alive		Dead		Total	
		N	%	N	%	N	%
Infraglottis	0	116	79,5%	7	50,0%	123	76,9%
Pathological aspect	1	30	20,5%	7	50,0%	137	23,1%
Total		146	100,0%	14	100,0%	160	100,0%

Chi-square test: (p) = 0.030 *

**Significance degree of involved subsites for total laryngectomy**

**Figure 3:**
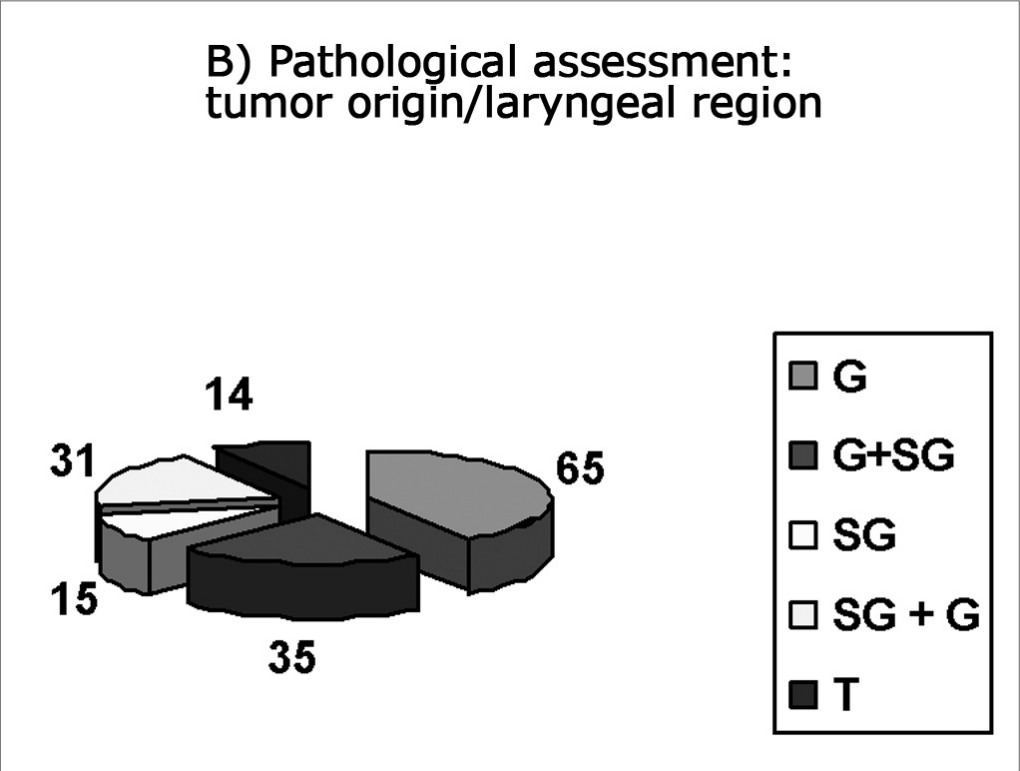

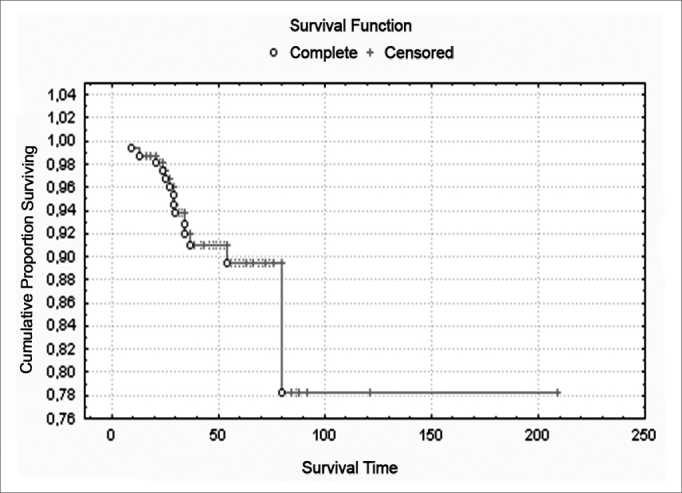
Survival curve from onset of symptoms to last visit - Has no legend.

Figure 3 **is a graphic representation of the larynx subsites.**

**Table:** 

Anterior commissure	Posterior commissure	Ventricle	Infraglottis
A) Tumors G	A) Tumors G	A) Tumors G	A) Tumors G
AC: (p) = 0.87	AC: (p) = 0.03	AC: (p) = 0.24	AC: (p) = 0.0007
PA: (p) = 0.09	PA: (p) = 0.0001	PA: (p) = 0.07	PA: (p) < 0.0001
B) Tumors G + SG	B) Tumors G + SG	B) Tumors G + SG	B) Tumors G + SG
AC: (p) = 0.48	AC: (p) = 0.22	AC: (p) = 1.000	AC: (p) = 0.29
PA: (p) = 0.84	PA: (p) = 0.19	PA: (p) = 0.96	PA: (p) = 0.04
C) Tumors SG	C) Tumors SG	C) Tumors SG	C) Tumors SG
AC: Test not applicable	AC: (p) = 0.37	AC: Test not applicable	AC: Test not applicable
PA: (p) = 0.46	PA: (p) = 1.000	PA: Test not applicable	AC: Test not applicable
D) Tumors SG+G	D) Tumors SG+G	D) Tumors SG+G	D) Tumors SG+G
AC: (p) = 1.00	AC: (p) = 0.73	AC: (p) = 0.36	AC: Test not applicable
PA: (p) = 1.00	PA: (p) = 0.67	PA: (p) = 0.34	AC: Test not applicable

**Key**:

G: Glottic tumors G+SG: Glottic tumors extending to the supraglottis

SG: Supraglottic tumors SG+G: Supraglottic tumors extending to the glottis

## KAPLAN MEIER SURVIVAL CURVE

The survival curve from onset of symptoms up to last visit is shown in Figure 4.

## DISCUSSION

In our study, the most frequent initial neoplasm site was the glottis (CA: 105; PA: 100). In transglottic tumors, the initial neoplasm site was also in the glottis of five patients in clinical assessment and of 10 patients in pathological assessment.

The main goal of the surgical treatment is to eradicate the primary tumor with free surgical margins, preserve the organ when necessary, in addition to cure or longer survival.

A total of 71 partial and 72 total laryngectomies were performed.

The subsites analyzed showed unique characteristics in terms of tumor invasion according to their anatomic formation.^1^,^2^,^8^,^12^

We started studying the subsites by the anterior commissure and its importance in indicating the surgical technique. The anterior commissure has important characteristics that should be taken into account in neoplasm dissemination, when approaching it surgically.^1^, ^2^, ^3^, ^4^, ^5^, ^6^, ^7^, ^8^, ^9^, ^10^, ^11^, ^12^

Its embryologic and anatomic formation has drawn attention of many authors, when studying laryngeal cancer dissemination.^8^ These formation aspects should be pointed out to indicate the open technique in initial tumors (T1 and T2). The main objective is tumor resection with free margins, and, consequently, local control of the disease.^2^,^4^,^6^, ^7^, ^8^, ^9^, ^10^, ^11^, ^12^ According to studies conducted by Rucci et al., the anterior commissure and the Broyles tendon have a clearly defined plane in which the blood vessel distribution is local, superficial, and restricted to the mucosa, with late maturation of these phonatory structures.^8^,^9^,^12^ This characteristic would provide slower dissemination of the neoplasm.^8^ The anterior commissure is considered a barrier by many authors.^2^,^6^,^7^ However, there is a weak point which is the insertion of the Broyles tendon, where there is no internal perichondrium, facilitating the inferior dissemination of supraglottic tumors and invasion of the cartilage.^2^,^7^,^8^ The approach of the anterior commissure in initial tumors has generated controversies in the literature regarding treatment.^5^,^9^,^17^ The authors supporting surgery suggest better local control and lower relapse for T1 and T2 tumors, with different results among the various surgical techniques.^4^,^8^,^10^,^11^ On the other hand, the supporters of radiation therapy emphasize local control similar to that obtained by surgery and better vocal quality.^5^

At the Escola Paulista de Medicina, after confirming anterior commissure involvement by the tumor, open surgery was indicated in most cases, except for five cases, in which endoscopic chordectomy was performed (four T1b patients and one T2 patient).

Sixty-seven patients underwent partial surgery with free margins. Two of them died due to local recurrence, the others are alive and disease-free. Four patients underwent conservative surgery and had involved margins. All four were referred to radiation therapy and one patient died because of the disease.

During the period studied, there was a clear tendency to approach the anterior commissure through open technique, especially through frontolateral laryngectomy for T1b (n=12) and T2 (n=9) tumors, and supracricoid laryngectomy for T2 (n=15) tumors, chiefly for those with decreased vocal fold mobility, and some T3 (n=6) tumors.

The results of 71 patients who underwent partial surgery were satisfactory, since only five patients had their surgeries converted to total laryngectomy; in that, four due to local relapse and one due to chronic aspiration. Two out of these five patients remained disease-free.

The study of the anterior commissure through partial surgeries was impaired due to insufficient number of procedures. However, the approach to the anterior commissure when treating initial tumors (T1b, T2) and rare T3 to preserve the larynx showed better results with the open technique.

The main objective of surgical and open approach is primary tumor resolution. The aim is to obtain free margins, local control with larynx preservation, regardless of higher or lower level of anterior commissure ionvolvement.^8^

In our study, greater involvement of anterior commissure in partial surgery procedures was found in tumors originating from or located in the glottis (G): CA: n=33 (75%), PA: n=24 (64.9%) and extending to the supraglottis (G+SG): CA: n=9 (40.9%), PA: n=11 (47.8%) and in supraglottic tumors extending to the glottis (SG+G): CA: n=10 (33.3%), PA: n=13 (100%).

On the other hand, in only supraglottic tumors (SG): CA: n=0 (0.0%), PA: n=6 (100.0%), the anterior commissure was less marked.

In regards to the sum of partial and total surgeries, the anterior commissure involvement was not significant for total laryngectomy in all tumors originated from or located in the glottis or supraglottis, as shown in the following results: (G): CA (p=0.87), PA (p=0.09); (G+SG): CA (p=0.48), PA (p=0.84); (SG): CA: non applicable; PA: (p=0.46) and (SG+G): CA (p= 1.000), PA: (p=1.000).

The presence of anterior commissure involvement in primary of secondary form in our patients with initial tumors showed better progression when open surgery was performed, in agreement with other authors.^4^,^8^, ^9^, ^10^, ^11^

The posterior commissure was significant for total laryngectomy in glottic tumors CA: (p=0.03) and PA: (p=0.0001). It is difficult to define the posterior commissure in terms of terminology, location, and components. Many authors disagree about the term posterior commissure, preferring to call it interarytenoid space or posterior glottic space. Rucci described it as a cartilaginous plane including the arytenoid medial surface, the vocal process, glands and blood vessels on the lamina propria, elastic lamina, and internal cricoarytenoid ligaments on both sides.^8^,^12^,^13^ Its structures are related to the sphincter function and it has an early maturation process. Consequently, it would make the neoplasm dissemination through the posterior commissure occur in a faster and more intense manner.8 Most tumors affecting the posterior commissure are located on the posterior two thirds of the vocal folds.^13^,^14^ The mucosa, in this site, is intimately attached to the arytenoids, and when the tumor reaches the thyroarytenoid muscle, it spreads through the paraglottic space, arytenoids, cricoarytenoid joint, and postcricoid region.^13^ Through the cricoarytenoid ligaments it quickly reaches the infraglottis.^13^ The tumor advance towards the cricoarytenoid joint and the infraglottis may render the conservative surgery unfeasible.^13^,^14^ This type of neoplasm evolution explain the greater involvement of the posterior commissure (G): CA: (p) = 0.03; PA: (p) = 0.0001 and of the infraglottis (G): CA: (p) = 0.0007; PA: (p) = 0.0001 and (G+SG): PA: (p) = 0.04, which in our study made the conservative surgery unfeasible, especially in glottic tumors. The infraglottis is a subsite that can generate false-negative1 results, during its assessment in an attempt to surgically preserve the larynx, as well as its prognosis in regards to survival, according to results presented.^1^,^15^ This is justified by the presence of neoplasm mucosal invasion in the fixed part of the infraglottis, where the superficial branches of the cricothyroid artery are found.^15^ These branches work as a means for diffusion, developing prelaryngeal, pretracheal, and/or paratracheal metastases, adjacent to the tracheostoma.^15^ This condition was reproduced in our patients, according to the records presented by the death rates in the pathological assessment (PA: (p) = 0.03).

The ventricle worked as an additional subsite in disseminating the tumor, showing an even more advanced stage of the disease, with no repercussion for radical treatment.

The predominance of patients with high posterior commissure and infraglottis involvement was well documented by the presence of T3 tumors (n=52) and T4 tumors (n=37), in which total laryngectomy demonstrated to be the best treatment for local control and for survival. Radical treatment, associated or not to radiation therapy for advanced tumors has brought similar results in regards to survival in the literature.^19^,^20^

Today there is a trend to treat T3 tumors to preserve the larynx, through organ preservation protocols, in addition to surgery, such as supracricoid laryngectomy.^18^,^19^,^21^ When surgical preservation is indicated, it is important to point out the difference between the fixation of vocal folds and of arytenoids.^16^,^21^ The first does not necessarily invalidate partial surgery, since the most frequent cause is invasion of the thyroarytenoid muscle, while the latter can mean involvement of the cricoarytenoid articulation, making it impossible to preserve the larynx.^13^,^16^ Laccourreye clearly demonstrated the feasibility of the articulation, and, therefore, the possibility of preserving the larynx with neoadjuvant chemotherapy, prior to the supracricoid laryngectomy.^20^,^21^

Clinical preservation was well supported through randomized protocols from the Veterans Affairs Laryngeal Cancer Study Group.^23^

Today, the results improved with concomitant chemo and radiation therapy regimens.^24^

Other studies with induction chemotherapy for locally advanced tumors showed larynx preservation in 44% of cases, with no increase in survival.^25^

Responses higher than 50% for T3 and T4 tumors through neoadjuvant chemotherapy can favor indications for supracricoid laryngectomy.^22^ However, for unsatisfactory responses or important damage to the larynx (T4), the management is total laryngectomy.^26^, ^27^, ^28^

Unfortunately, in our study, patients (T3 and T4 tumors) referred to clinical treatment did not progress well. Only five patients with tumors (T3) stayed alive with no carcinoma; the others died from the disease.

Lefebvre had already pointed out that the anatomic preservation of the larynx through organ preservation protocols would not always mean a functional larynx.^3^

The satisfactory results obtained by our patients through the Kaplan Meier curve are due to appropriate surgical treatment, both in conservative and total surgery, to achieve free margins. The indication of the surgical technique was according to staging and degree of involvement of subsites, to reach local control and better survival.

## CONCLUSIONS

The involvement of the anterior commissure, especially in initial tumors (T1, T2) and rare T3 with possible surgical preservation of the larynx is better treated with open surgery. Hence, frontolateral laryngectomy and supracricoid laryngectomy showed to be effective in obtaining free surgical margins, local control and organ preservation in initial tumors and T3 tumors selected, regardless if the level of anterior commissure involvement is primary or secondary.

The ventricle represents an additional site in disseminating the disease.

The greater involvement of the posterior commissure and infraglottis require total laryngectomy for being the most suitable treatment to obtain better locoregional control. However, the degree of involvement of the infraglottis may compromise survival even when free margins are present, in total laryngectomies.
